# Next-Generation Prosthetic Hand: from Biomimetic to Biorealistic

**DOI:** 10.34133/2021/4675326

**Published:** 2021-03-24

**Authors:** Ning Lan, Manzhao Hao, Chuanxin M. Niu, He Cui, Yu Wang, Ting Zhang, Peng Fang, Chih-hong Chou

**Affiliations:** ^1^Institute of Medical Robotics, Shanghai Jiao Tong University, Shanghai, China; ^2^Laboratory of Neurorehabilitation Engineering, School of Biomedical Engineering, Shanghai Jiao Tong University, Shanghai, China; ^3^Department of Rehabilitation Medicine, Ruijin Hospital, School of Medicine, Shanghai Jiao Tong University, Shanghai, China; ^4^Center for Excellence in Brain Science and Intelligent Technology, Chinese Academy of Sciences, Shanghai, China; ^5^School of Biological Science and Medical Engineering, Beihang University, Beijing, China; ^6^Beijing Advanced Innovation Center for Biomedical Engineering, School of Biological Science and Medical Engineering, Beihang University, Beijing, China; ^7^i-lab, Key Laboratory of Multifunctional Nanomaterials and Smart Systems, Suzhou Institute of Nano-Tech and Nano-Bionics, Chinese Academy of Sciences, Suzhou, China; ^8^Shenzhen Institute of Advanced Technology, Chinese Academy of Sciences, Shenzhen, China

## Abstract

Integrating a prosthetic hand to amputees with seamless neural compatibility presents a grand challenge to neuroscientists and neural engineers for more than half century. Mimicking anatomical structure or appearance of human hand does not lead to improved neural connectivity to the sensorimotor system of amputees. The functions of modern prosthetic hands do not match the dexterity of human hand due primarily to lack of sensory awareness and compliant actuation. Lately, progress in restoring sensory feedback has marked a significant step forward in improving neural continuity of sensory information from prosthetic hands to amputees. However, little effort has been made to replicate the compliant property of biological muscle when actuating prosthetic hands. Furthermore, a full-fledged biorealistic approach to designing prosthetic hands has not been contemplated in neuroprosthetic research. In this perspective article, we advance a novel view that a prosthetic hand can be integrated harmoniously with amputees only if neural compatibility to the sensorimotor system is achieved. Our ongoing research supports that the next-generation prosthetic hand must incorporate biologically realistic actuation, sensing, and reflex functions in order to fully attain neural compatibility.

## 1. Background

Modern prosthetic hands mimic the anatomical structure and appearance of human hand but miss the biological underpinnings of actuation and sensing, thus lacking the similar functionality of human sensorimotor system. Many amputees choose to abandon prosthetic hands due to difficulties in control and absence of tactile awareness while grasping objects [1]. Sophisticated prosthetic hand often does not perform better than a simple gripper actuated by contralateral shoulder through a cable, which mediates an extended physiological proprioception (EPP) [2]. Rigid motor actuation does not lend prosthetic hand with compliance for grasping soft or fragile objects.

Humans have developed effective strategies for dexterous control of hand grasps without detailed motion planning at the actuator/effector levels. The biological mechanisms arise from compliant actuation of muscles and rich sensory afferents from proprioceptors and cutaneous receptors. These advantages of biological sensorimotor control allow an adaptive adjustment of hand stiffness at the fingers to that of the object grasped without precisely planning finger movements. Here, we advance a view that restoring biologically realistic compliant actuation and tactile sensory feedback in prosthetic hand may transform prosthetic grasp functions [3–7].

## 2. Human Sensorimotor Control

**Figure 1:**
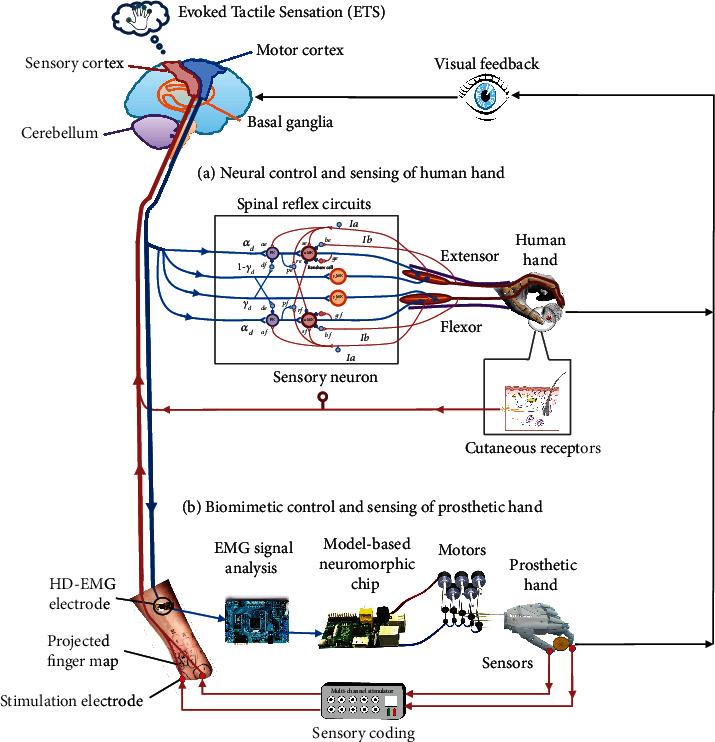
Illustration of neural control of a human hand (a) and biomimetic control of a prosthetic hand (b). Before amputation, the brain controls human hand through an intact neuromuscular reflex system along with proprioceptive and cutaneous sensory afferents. In prosthetic control, the neuromuscular reflex process is amiss, and the sensory feedback information provided may be limited and incompatible to what is acquainted to the brain. Thus, it is essential to restore the neuromuscular reflex process and natural sensory feedback for prosthetic hand. Visual information determines hand positioning and opening. Abbreviations: HD-EMG: high-density EMG; ETS: evoked tactile sensation. Part (a) is modified from Figure 2 in [10] with permission.

Human sensorimotor control serves as a perfect model for prosthetic hands. The hierarchical metaphor of biological motor control as shown in Figure 1(a) may help to bridge the gap of understanding of motor control between human and robotics/prosthetics. The top level in the brain programs gross aspects of movements at the joint or endpoint of limbs. Movements are then translated into specific motor commands to muscles at the spinal level by muscle synergies [8]. Interneurons, particularly the propriospinal neurons (PN) [9, 10], in the spinal cord may form such a module of muscle synergies. At the bottom level, motor commands are executed by muscles acting at joints of limbs. The brain is well informed by proprioceptors and skin receptors that monitor the process and outcome of hand grasp. However, amputation disrupts both efferent and afferent sensorimotor signals. Achieving biological actuation, sensing, and control like human sensorimotor system entails restoring the disrupted peripheral sensorimotor processes.

Muscles possess highly nonlinear biomechanics with force dependent on fascicle length and velocity of contraction, which gives rise to the desirable compliance with stiffness and viscosity. Muscle viscoelasticity is further regulated by local reflex circuits at the *α*-motoneuron pools of the spinal cord [11]. It allows the brain to maintain equilibrium positions of limbs. A compliant limb is able to cope with unexpected disturbances during movement or posture, so that the brain does not need to compute explicitly forces and trajectories of the limb.

Muscles are embedded with proprioceptors, i.e., spindles and Golgi tendon organs (GTO), that are sensitive to muscle stretch and force [12]. The spindles are innervated by numerous *γ*-static and *γ*-dynamic motoneurons, which modulate spindle sensitivity. *γ* motoneurons are comodulated with *α* motoneurons to update peripheral muscles about kinematics of programmed movement by the brain [13].

Vision computes the locations of static or moving objects in space, which is used by the brain for planning and directing sensorimotor actions in reaching and grasp. Proprioceptive afferents inform the brain about muscle states and limb positions during execution of motor plans [12]. Tactile information from skin receptors allows the brain to discern physical attributes of grasped objects. It provides the brain with multimodalities of senses, such as touch, pressure, texture, temperature, and pain, so that human can manipulate soft or sharp objects effectively. Without tactile sensation, the hand must be placed under closed-loop control of visual supervision. Therefore, it is essential to restore neuromuscular reflex and tactile sensation in amputees, whose limb muscles are partially available and tactile sensation from hand is totally lost.

## 3. Biorealistic Approach with Emerging Technologies

The major flaw in current prosthetic system compared with human limbs is that the efferent motor information from the brain and afferent sensory information to the brain are interrupted, and actuators of prosthetic limb are electric motors without the compliant flexibility of human neuromuscular system. A variety of implantable technologies have been developed to restore the functions of nervous system [3, 4, 7, 14, 15]. We are focusing on developing noninvasive and biorealistic technologies for the next-generation hand prosthesis.  Figure 1(b) illustrates a potential biorealistic approach to developing the next-generation prosthetic hand. The goal is to improve the neural compatibility [16] by restoring the disrupted neuromuscular reflex and tactile sensation. These emerging technologies include the following:

- Generative BMI models of motor signal decoding: a generative brain-machine interface (BMI) model can provide a feedforward control strategy for more biomimetic and flexible neuroprosthetic control for reaching and grasping [17, 18]. This technology is in evaluation in nonhuman primates.
- Recognition of motor intention using high-density EMG: novel pattern recognition methods can improve predicting motor intentions from high-density electromyogram (EMG) signals of residual muscles in amputated limb, which enables an intuitive control of prosthetic movements [19]. This technology has been applied to amputee control of prosthetic hand.
- Neuromorphic models of neuromuscular system: it is feasible to restore neuromuscular reflex process for cable-driven prosthetic hands with human-like traits, using neuromorphic hardware to perform fast computation of physiologically realistic models and to emulate human-like reflex in real time [6]. This technology is now in evaluation for human application.
- Noninvasive tactile sensory feedback: we have demonstrated the feasibility to utilize an electrically evoked tactile sensation (ETS) as a natural way to furnish sensory information to amputee's brain. This sensory feedback technique is noninvasive, has long-term stability, and can supply finger-specific, multiple modalities of natural sensory information to amputees [5, 20]. This technology is currently undergoing evaluation of functional benefits in amputee subjects.
- Selective slip sensors mimicking the Ruffini endings: a flexible tactile sensor inspired by the structure of fingerprints and the function of the Ruffini endings with selective sensitivity to static and sliding friction forces may enable prosthetic hand to handle slippage during grasping [21]. The proof of concept of this technology is completed. The device is in the initial stage for human application.
- Tendon-driven soft prosthetic hand: a tendon-driven soft prosthetic hand with finger body made of continuum spiral structure of super-elastic nitinol alloy material can have natural compliance for grasping objects using a simple control strategy [22]. This device is in refinement of structural design and functional evaluation.

## 4. Conclusion and Perspective

Developing a prosthetic hand with biorealistic elements of human sensorimotor system may enhance its neural compatibility [16]. An incompatible prosthetic hand may result in reduced functionality or rejection. Past research has accumulated a large body of knowledge on human sensorimotor system [9, 23–26]. It is now technologically mature to translate this body of knowledge to design biorealistic prosthetic hands that restore neuromuscular mechanics [6], spinal reflexes [13], and tactile feedback [3–5, 7]. This may result in not only superior performance but also a new generation of prosthetic hands. Understanding neural compatibility between prosthetic hand and human sensorimotor system may broadly impact the design of prosthetic and therapeutic devices [16]. The biorealistic approach should also facilitate reverse engineering to understand neural mechanisms of brain control of movements in humans [27].
